# The CF-Sputum Induction Trial (CF-SpIT) to assess lower airway bacterial sampling in young children with cystic fibrosis: a prospective internally controlled interventional trial

**DOI:** 10.1016/S2213-2600(18)30171-1

**Published:** 2018-06

**Authors:** Katherine Ronchetti, Jo-Dee Tame, Christopher Paisey, Lena P Thia, Iolo Doull, Robin Howe, Eshwar Mahenthiralingam, Julian T Forton

**Affiliations:** a Department of Paediatric Respiratory Medicine, Noah's Ark Children's Hospital for Wales, Cardiff, UK; b Department of Paediatric Physiotherapy, Noah's Ark Children's Hospital for Wales, Cardiff, UK; c School of Biosciences, Cardiff University, UK; d School of Medicine, Cardiff University, UK; e Department of Microbiology, University Hospital for Wales, Cardiff, UK

## Abstract

**Background:**

Pathogen surveillance is challenging but crucial in children with cystic fibrosis—who are often non-productive of sputum even if actively coughing—because infection and lung disease begin early in life. The role of sputum induction as a diagnostic tool for infection has not previously been systematically addressed in young children with cystic fibrosis. We aimed to assess the pathogen yield from sputum induction compared with that from cough swab and single-lobe, two-lobe, and six-lobe bronchoalveolar lavage.

**Methods:**

This prospective internally controlled interventional trial was done at the Children's Hospital for Wales (Cardiff, UK) in children with cystic fibrosis aged between 6 months and 18 years. Samples from cough swab, sputum induction, and single-lobe, two-lobe, and six-lobe bronchoalveolar lavage were matched for within-patient comparisons. Primary outcomes were comparative pathogen yield between sputum induction and cough swab for stage 1, and between sputum induction, and single-lobe, two-lobe, and six-lobe bronchoalveolar lavage for stage 2. Data were analysed as per protocol. This study is registered with the UK Clinical Research Network (14615) and with the International Standard Randomised Controlled Trial Network Registry (12473810).

**Findings:**

Between Jan 23, 2012, and July 4, 2017, 124 patients were prospectively recruited to the trial and had 200 sputum induction procedures for stage 1. 167 (84%) procedures were successful and the procedure was well tolerated. Of the 167 paired samples, 63 (38%) sputum-induction samples were pathogen positive compared with 24 (14%) cough swabs (p<0·0001; odds ratio [OR] 7·5; 95% CI 3·19–17·98). More pathogens were isolated from sputum induction than cough swab (79 [92%] of 86 *vs* 27 [31%] of 86; p<0·0001). For stage 2, 35 patients had a total of 41 paired sputum-induction and bronchoalveolar lavage procedures. Of the 41 paired samples, 28 (68%) were positive for at least one of the concurrent samples. 39 pathogens were isolated. Sputum induction identified 27 (69%) of the 39 pathogens, compared with 22 (56%; p=0·092; OR 3·3, 95% CI 0·91–12·11) on single-lobe, 28 (72%; p=1·0; OR 1·1, 95% CI 0·41–3·15) on two-lobe, and 33 (85%; p=0·21; OR 2·2, 95% CI 0·76–6·33) on six-lobe bronchoalveolar lavage.

**Interpretation:**

Sputum induction is superior to cough swab for pathogen detection, is effective at sampling the lower airway, and is a credible surrogate for bronchoalveolar lavage in symptomatic children. A substantial number of bronchoscopies could be avoided if sputum induction is done first and pathogens are appropriately treated. Both sputum induction and six-lobe bronchoalveolar lavage provide independent, sizeable gains in pathogen detection compared with the current gold-standard two-lobe bronchoalveolar lavage. We propose that sputum induction and six-lobe bronchoalveolar lavage combined are used as standard of care for comprehensive lower airway pathogen detection in children with cystic fibrosis.

**Funding:**

Health and Care Research Wales—Academic Health Science Collaboration and Wellcome Trust Institutional Strategic Support Fund.

## Introduction

Longitudinal surveillance studies using repeated bronchoalveolar lavage in children with cystic fibrosis have reported that 30% of these children have *Pseudomonas aeruginosa* detected in the first 6 years of life,^1^ and that infection with significant pathogens occurs in the first 2 years of life in 71% of children.^2^ Notably, early infection was identified as the major determinant of lung function deterioration by school age, suggesting that it is an important driver of lung inflammation and has a crucial contribution to the development of cystic fibrosis lung disease.^2^ Young children with cystic fibrosis are generally asymptomatic, cough free, and non-productive of mucus. These children are often incapable of expectorating sputum even if actively coughing during an exacerbation. Effective sampling for lower airway microbiology is therefore problematic, yet remains crucial in this age group if infection is to be effectively treated or prevented, and the potential benefits of newborn screening properly realised.^3^ Cystic fibrosis standards of care recommend doing regular oropharyngeal cough swabs for bacterial surveillance in young non-expectorating children. However, oropharyngeal cultures are a poor surrogate for cultures from lower airway samples taken at concurrent bronchoalveolar lavage.^4^

**Research in context**

**Evidence before this study**

We initially did a comprehensive review of the use of sputum induction in children with cystic fibrosis on March 1–15, 2011. We searched PubMed for studies published between Jan 1, 1960, and Dec 31, 2010, and updated the search on Dec 6, 2014. We used the following keywords: “induced sputum”, “sputum induction”, “bronchoalveolar lavage”, “cough swab”, “oropharyngeal”, “children”, “child”, “infant” “childhood”, “young”, “cystic fibrosis”, “CF”, and “hypertonic saline”. Few adequately powered studies were found. Six studies assessed sputum induction in children with cystic fibrosis and these were generally in older children who could perform spirometry reliably. Taken together, these studies included 211 patients and reported a 92·5% success rate in obtaining a sputum sample. Four studies compared sputum induction with oropharyngeal samples in children with cystic fibrosis. The two larger studies identified additional organisms on sputum induction in 30% and 42% of cases, but these studies mainly recruited school-age children and teenagers, many of whom could spontaneously expectorate sputum. One small study compared sputum induction with bronchoalveolar lavage in ten children with cystic fibrosis. During the period of recruitment to the present study, one study was published that showed sputum induction to be superior to oropharyngeal sampling in 32 children younger than 5 years, with an approximately two-fold increase in pathogen detection. A further single study compared sputum induction to gold-standard two-lobe bronchoalveolar lavage in children, but paired samples were not necessarily taken at the same visit. That study found sputum induction sensitivity to be 37% and specificity to be 69%, compared with gold-standard two-lobe bronchoalveolar lavage, but discounted any pathogens isolated exclusively on sputum induction as false negatives.

**Added value of the study**

To our knowledge, this is the first time a study has compared concomitant cough swab, sputum induction, and the gold-standard two-lobe bronchoalveolar lavage to comprehensive six-lobe bronchoalveolar lavage to systematically define the relative contribution of each approach. Our data establish sputum induction as superior to cough swab and as a credible approach to sampling the lower airway in symptomatic children with cystic fibrosis when compared with bronchoalveolar lavage. This study shows that both sputum induction and six-lobe bronchoalveolar lavage contribute independent, sizeable gains in pathogen detection over and above two-lobe bronchoalveolar lavage, and challenges two-lobe bronchoalveolar lavage as an adequate gold-standard approach to understanding lower airway microbiology. Sputum induction is uniquely placed to sample the large intrathoracic airways, a compartment inadequately considered in the current paradigm of lower airway sampling. In symptomatic patients, doing sputum induction before bronchoalveolar lavage will correctly describe the lower airway pathogen environment in most patients, thereby markedly reducing the number of bronchoscopy procedures required.

**Implications of all available evidence**

Our study supports the recommendation that sputum induction and six-lobe lavage should be done together as a new standard of care for comprehensive assessment of the lower airway pathogen environment in children with cystic fibrosis. If sputum induction is done before bronchoalveolar lavage, a substantial number of bronchoscopies could be avoided in symptomatic children with cystic fibrosis. The inclusion of sputum induction as a regular contribution to cystic fibrosis care in children is supported by the tolerability of the procedure in all age groups, the ease of repeatability, the acceptability to parents and patients, the high success rates, the additional pathogens identified, and the clear economic savings.

Bronchoalveolar lavage is considered to be the gold standard for sampling lower airway microbiology.^5^ Although the international community is interested in bronchoalveolar lavage-based microbiology-surveillance programmes, little evidence supports recommendation of this invasive approach in routine cystic fibrosis care.^6^, ^7^ Bronchoalveolar lavage is generally reserved for children with cystic fibrosis who have not responded to appropriate or empirical antibiotic treatment and in whom oropharyngeal cultures do not offer an explanation for the persistence of symptoms. No consensus exists on methods for bronchoalveolar lavage, and practice varies. Guidelines for children with cystic fibrosis recommend two-lobe bronchoalveolar lavage taken as follows: three-aliquot bronchoalveolar lavage from the right-middle lobe and a single-aliquot bronchoalveolar lavage from the lingular or the most affected lobe.^5^ A study published in 2011^8^ showed comprehensive six-lobe bronchoalveolar lavage sampling to be safe, well tolerated, and superior to single-lobe^9^ or two-lobe^5^ bronchoalveolar lavage, suggesting that bacterial communities might be compartmentalised within the lung.

Clinically, young children with cystic fibrosis have wet bronchitic-type coughs during infection, suggesting the predominant focus of infection might be the large intrathoracic airways rather than the alveolar bed. The large intrathoracic airway compartment is not routinely sampled because it is largely bypassed by even the most extensive approach to bronchoalveolar lavage. Sputum induction is a safe approach to obtaining lower airway samples from patients who are not spontaneously productive^10^, ^11^ and its use in tuberculosis surveillance in children is well established. The role of sputum induction in the care of young children with cystic fibrosis has not been systematically addressed and few studies exist.^12^, ^13^, ^14^, ^15^, ^16^ Sample size and patient age have been variable in these studies but for the most part, conclusions have been encouraging. This trial (the Cystic Fibrosis Sputum Induction Trial [CF-SpIT]) takes a systematic approach to comprehensively investigate and compare bacterial sampling techniques in young children with cystic fibrosis.

We aimed to test sputum induction as an infection diagnostic for bacterial sampling in children with cystic fibrosis compared with concurrent standard cough swab, single-lobe bronchoalveolar lavage, the gold-standard two-lobe bronchoalveolar lavage, and also comprehensive six-lobe bronchoalveolar lavage.

## Methods

### Study design and participants

Cf-SpIT is a prospective internally controlled interventional single-centre trial done at the Children's Hospital for Wales (Cardiff, UK) in children with cystic fibrosis, comparing sputum induction, as a diagnostic intervention for pathogen detection, with concurrent cough swab, single-lobe bronchoalveolar lavage, gold-standard two-lobe bronchoalveolar lavage, and comprehensive six-lobe bronchoalveolar lavage. The study was subject to Institutional Review by the Cardiff and Vale Research Review Service (CaRRS—Project ID 11-RPM-5216) and approved by the South Wales Research Ethics Committee (11/WA/0334).

We prospectively recruited children with cystic fibrosis aged between 6 months and 18 years, from the South, West and Mid-Wales Children's Cystic Fibrosis Network. All children attending the Children's Hospital for Wales for clinically indicated bronchoscopy, those attending for routine surgery under general anaesthetic, those admitted for treatment of a chest exacerbation, or those attending for annual review in the outpatient clinic were eligible. Children on treatment antibiotics at the time of sampling were excluded, to maximise the chances of successful bacterial culture.

The study was structured in two stages, each designed to test different hypotheses. In stage 1, sputum induction as a diagnostic intervention was tested against cough swab. Children were recruited for this stage of the study at any point when they would otherwise have a cough swab taken: in the outpatient clinic or as an inpatient before receiving intravenous antibiotics. In stage 2, sputum induction as a diagnostic intervention was tested against bronchoalveolar lavage. This was done in a subgroup of patients who had been recruited into stage 1, and who were also attending for a clinically indicated bronchoscopy and bronchoalveolar lavage. Specifically, sputum induction was compared to single-lobe bronchoalveolar lavage, two-lobe bronchoalveolar lavage, and six-lobe bronchoalveolar lavage.

Children could volunteer to take part on more than one occasion if the occasions were more than 3 months apart. Sputum induction was done immediately after cough swab and within the 24 h before bronchoscopy if paired with bronchoalveolar lavage. Children were classified as symptomatic at the time of recruitment if they had an increase in respiratory symptoms, defined as a wet or dry cough, wheeze, or coryzal symptoms. The cough was defined as wet if it sounded wet before the procedure. Data on pathogen isolates from the preceding 12 months before the procedure and new treatments commenced because of microbiology results from the procedure were recorded for all children.

Informed consent was taken by trained clinicians on the delegation log. Data for recruitment, clinical observations, and primary and secondary outcome measurements were collated by trained clinicians and research staff on the delegation log and managed by the chief investigator. Regular progress and safety reports were submitted to Research and Design and Ethics panels by the chief investigator.

### Procedures

Sputum induction was done by a specialist physiotherapist. 8 mL of 7% hypertonic sodium chloride solution was administered through a simple disposable oxygen-driven jet nebuliser set (SideStream disposable kit; Philips Respironics, Murrysville, PA, USA) at 5 L/min for 15 min and physiotherapy was given during and after the nebuliser was completed. A combination of percussion, vibration, positive expiratory pressure, and active cycle of breathing compatible with the patient's normal physiotherapy regimen was used. Oropharyngeal suction using a size 6, 8, or 10F suction catheter was used to obtain a sputum sample in children who could not spontaneously expectorate after the procedure. Duration of the procedure was limited to 30 min. Success of the procedure was defined as the ability to obtain a mucoid sample, per visual inspection. The physiotherapist documented heart rate, respiratory rate, and FEV_1_ where applicable before and after the procedure as objective measures of tolerance (appendix).

All bronchoscopy was done under general anaesthetic. Suction of secretions was avoided before bronchoalveolar lavage to preserve localised sampling without contamination. Samples were taken from all six lobes specifically in the following order: right middle lobe (RML), left lingular (LLi), right lower lobe (RLL), right upper lobe (RUL), left lower lobe (LLL), and left upper lobe (LUL). RML bronchoalveolar lavage was done using three aliquots of 1 mL/kg normal saline (maximum 20 mL per aliquot) with low-level suction through the scope used to retrieve the sample between aliquots. A single aliquot of 1 mL/kg bodyweight (maximum 20 mL) was used for all other lobes. This aliquot was retrieved by light suction on the syringe used for instillation before the liquid column was broken. A second instillation of 1 mL/kg (maximum 20 mL) was used for all lobes in which the initial aliquot did not return 40% volume or greater using syringe back-suction.

Bronchoalveolar lavage fluid from each individual was processed as three samples. All three aliquots from the RML were combined to form bronchoalveolar lavage sample 1. The single aliquot from the LLi was used as sample 2. Fluids from the remaining four lobes (RLL, RUL, LLL, and LUL) were combined to form sample 3. These three samples were sent to the microbiology lab where they were processed independently.

Because bronchoalveolar lavage samples 1, 2, and 3 were taken in strict sequential order in all bronchoscopy procedures, we were able to combine the pathogens isolated to describe pathogen detection from increasingly extensive bronchoalveolar lavage. Pathogens isolated from sample 1 were used to describe pathogen detection from single-lobe bronchoalveolar lavage; pathogens isolated from samples 1 and 2 were combined to describe pathogen detection from two-lobe bronchoalveolar lavage; and pathogens isolated from samples 1, 2, and 3 were combined to describe pathogen detection from six-lobe bronchoalveolar lavage.

Airway samples were processed using standard techniques for bacteria and fungi in the hospital laboratory of the University Hospital of Wales. The laboratory uses standards from the CF Trust Guidelines.^17^ For all samples, *Haemophilus influenzae, Staphylococcus aureus*, meticillin-resistant *S aureus* (MRSA), *P aeruginosa, Burkholderia cepacia* complex species, non-tuberculous *Mycobacterium* species*, Achromobacter xylosoxidans, Stenotrophomonas maltophilia*, and *Klebsiella pneumoniae* were defined as cystic fibrosis airway pathogens.

All airway fluid samples were divided and one aliquot frozen at −80°C within 30 min of collection. Batch DNA extraction was done after a single freeze thaw, in an extraction-dedicated containment level 2 laboratory. Ribosomal intergenic spacer analysis (RISA) was done as described previously (appendix).^18^, ^19^

### Outcomes

The primary outcome was pathogen detection for all comparisons, measured by the proportion of patients with one or more positive samples (pathogen positive) and the number of pathogens isolated by each sampling approach. Secondary outcomes were success of sputum induction, proportion of cases in which sputum induction resulted in a change of treatment, test-specific detection rates for all approaches against all pathogens isolated, and the sensitivity of each sampling approach to correctly identify all pathogens isolated from the lower airway.

Subjective tolerance was assessed using visual analogue Likert-type scales (score 1–10)^20^ completed by the parent or child, or both, and the physiotherapist who did the procedure.

### Statistical analysis

We used discordant proportions to compute sample size. At the time of study initiation, no data were available on pathogen yield from sputum-induction sampling in children younger than 6 years with cystic fibrosis. Al-Saleh and colleagues^13^ studied sputum induction and throat swabs in 94 older children (mean age 12·1 years) with cystic fibrosis, most of whom could spontaneously expectorate. Discrepant culture results were seen in 27% of paired samples. For stage 1, assuming sputum induction would be less successful in the younger age group who could not spontaneously expectorate, we powered the study to detect a smaller discrepancy of 20% in culture results between cough swab and sputum induction. We used discordant proportions in keeping with the findings from Al-Saleh and colleagues^13^ (27% discordance, odds ratio [OR] 8). Using these proportions, we calculated that a sample size of 59 pairs is capable of detecting 20% discordance in culture results with a power of 80% and probability of type I error of 0·05.

We planned subgroup analyses by age (<6 years or ≥6 years) and symptom status (asymptomatic or symptomatic) for the stage 1 comparison of cough swab versus sputum induction. Assuming the same discordant proportions for all age groups, we required a sample size of 59 for each age subgroup. We therefore continued recruiting until at least 59 patients were recruited to each age subgroup, estimating that, taking patient withdrawals and exclusions into account, this would require prospective recruitment of 200 children in total.

No data comparing sputum induction with bronchoalveolar lavage were available at the time of study initiation. Therefore, for stage 2, we estimated discordant proportions to calculate the sample size. We estimated that a small proportion of pathogens (5%) would be isolated on sputum induction and not on bronchoalveolar lavage, and estimated discordance in culture results at 34%. A sample size of 44 pairs would be able to detect a 34% discrepancy between sputum induction and bronchoalveolar lavage with a power of 80% and type I error of 0·05.

We compared all paired proportions between sampling techniques using the two-sided McNemar's test. We used binary logistic regression (BLR) to assess the effect of repeated measurements within the cohort on the success rate of sputum induction and on the rate of pathogen positivity of sputum induction. We used BLR to assess the effect of age, the presence of respiratory symptoms, the ability to expectorate spontaneously before the procedure, and the need for oropharyngeal suction during the procedure, on the number of pathogens detected by sputum induction, using generalised estimating equations (GEE)^21^ to account for correlation between repeated measurements in the same individual.

We used test-specific detection rates when comparing different approaches to sampling, to interrogate the relative pathogen yield and help understand the relative sampling ability of each approach.

We generated a sensitivity analysis for each sampling approach against a combined gold standard consisting of all pathogens isolated from sputum induction and six-lobe bronchoalveolar lavage. A positive outcome was defined as the ability to identify all pathogens from the combined gold standard. This enabled us to quantify the ability of any single approach to correctly detect all lower airway pathogens in a given patient. We used BLR to assess the effect of age and the presence of respiratory symptoms on the ability of sputum induction to correctly detect all lower airway pathogens, using GEE^21^ to account for correlation between repeated measurements in the same individual. Statistical analyses were done using SPSS statistics for Windows, version 23.0. This study is registered with the UK Clinical Research Network (14615) and with the International Standard Randomised Controlled Trial Network Registry (12473810).

### Role of the funding source

The funder of the study had no role in study design, data collection, data analysis, data interpretation, or writing of the report. JTF, KR, and J-DT had access to the raw data. The corresponding author had full access to all of the data and the final responsibility to submit for publication.

## Results

**Figure 1:**
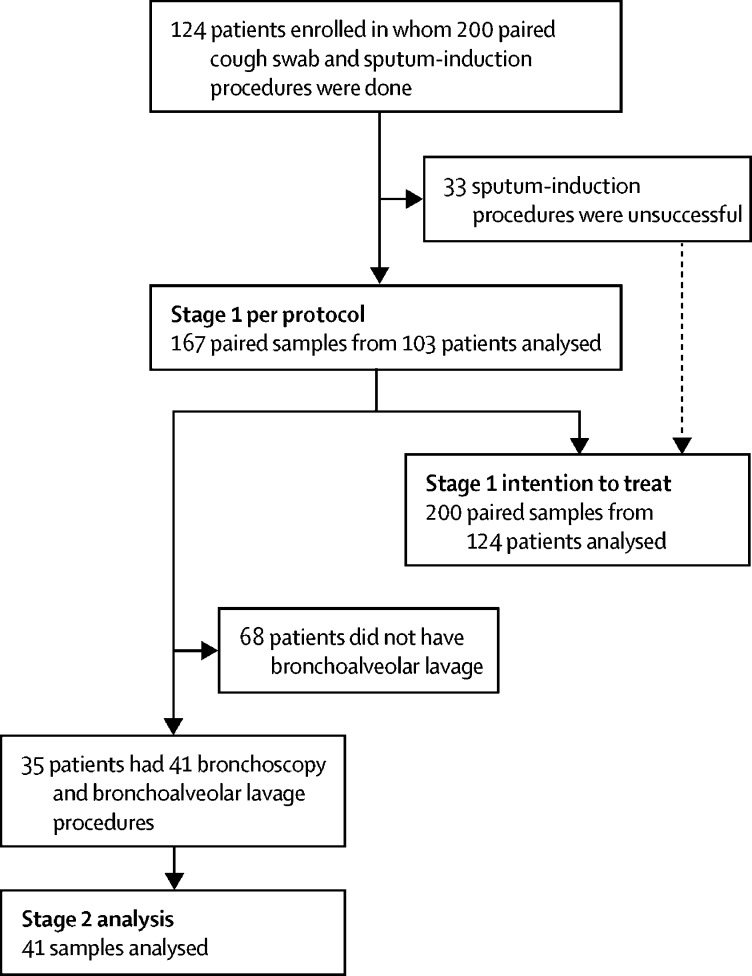
Participant flow diagram Patients could contribute a sample to the trial on more than one occasion if samples were taken at least 3 months apart.

Between Jan 23, 2012, and July 4, 2017, 124 patients were prospectively recruited to the trial and had 200 sputum induction procedures (figure 1; table 1). Median time between procedures in patients that contributed more than one sample was 12 months (IQR 5·5–19). 72 (36%) of the 200 procedures were done in children younger than 6 years and 128 (64%) were done in children aged 6 years or older. 128 (64%) of the samples came from children who were symptomatic at the time of sampling.

**Table 1:** Patient baseline characteristics

	**Stage 1: sputum induction**	**Stage 2: bronchoalveolar lavage**
Number of patients recruited	124	35
Number of procedures	200	41
Median age at procedure	8·2 years (4·9–12·6)	8·5 years (6·5–12·6)
Number of procedures in children aged <6 years	72 (36%)	..
Median age (subgroup <6 years)	3·5 years (1·6–4·9)	..
Number of procedures in children aged ≥6 years	128 (64%)	..
Median age (subgroup ≥6 years)	11·1 years (8·2–14·3)	..
*Pseudomonas aeruginosa* positive (isolated in preceding 12 months)	24 (12%)	6 (15%)
Median FEV_1_ (where applicable)	89% (76–99)	84% (72–94)
Hypertonic saline naive	37 (19%)	3 (7%)
Wet cough at time of procedure	66 (33%)	14 (34%)
Able to spontaneously expectorate before procedure	22 (11%)	2 (5%)
Symptomatic at time of procedure	128 (64%)	32 (82%)
Inpatient procedure	80 (40%)	41 (100%)
Outpatient procedure	120 (60%)	0

Data are n (%) or median (IQR). Denominators for percentages are number of procedures.

167 (84%) of 200 sputum-induction procedures were successful in producing a mucoid sputum sample. Repeated measures in those individuals recruited more than once did not affect the success of sputum induction (p=0·53). 22 (11%) of 200 children were able to expectorate sputum spontaneously before the sputum-induction procedure. 87 (44%) of 200 children could expectorate sputum during the procedure without requiring suction. When analysed by age group, sputum induction was equally as successful in children younger than 6 years (62 [86%] of 72) as in those aged 6 years or older (105 [82%] of 128). Age as a continuous variable did not influence the success of the sputum-induction procedure (p=0·55). However, oral suction was required in 56 (90%) of 62 children younger than 6 years versus 24 (23%) of 105 children aged 6 years or older. The sputum-induction procedure was similarly successful in the inpatient versus outpatient setting (100 [84%] of 120 *vs* 67 [83%] of 80), in symptomatic versus asymptomatic children (110 [86%] of 128 *vs* 57 [79%] of 72), in those with a wet versus dry cough (59 [89%] of 66 *vs* 108 [81%] of 134), and in patients who were naive to hypertonic saline versus those who were not (33 [89%] of 39 *vs* 134 [82%] of 169).

Of the 167 paired cough swab and sputum-induction samples, 63 (38%) sputum-induction samples were pathogen positive compared with 24 (14%) cough swabs (p<0·0001; OR 7·5; 95% CI 3·19–17·98). Repeated measures in individuals who gave more than one sample did not affect pathogen positivity in sputum-induction samples (p=0·99). An intention-to-treat analysis (ITT) in which unsuccessful sputum-induction attempts were classified as negative results remained significant (p<0·0001).

In subgroup analysis by age, in children younger than 6 years, 18 (29%) of 62 sputum-induction samples were pathogen positive compared with eight (13%) of 62 cough swabs (p=0·021; ITT analysis p=0·049). In children aged 6 years or older, 45 (43%) of 105 sputum-induction samples were pathogen positive compared with 16 (15%) of 105 cough swabs (p<0·0001; ITT analysis p<0·0001). Age as a continuous variable was significant in predicting whether sputum would be pathogen positive (p=0·0028), whereas the ability to expectorate spontaneously before the procedure was not predictive (p=0·86). The ability to expectorate during the procedure did not show an independent effect over age on whether sputum would be pathogen positive (p=0·24).

Sputum induction was more likely to be pathogen positive than cough swab in symptomatic children (46 [42%] of 110 *vs* 16 [15%] of 110; p<0·0001) and in asymptomatic children (17 [30%] of 57 sputum-induction samples vs eight [14%] of 57 cough swabs; p=0·049). The likelihood of sputum induction being pathogen positive was not significantly affected by whether the child was symptomatic or asymptomatic (p=0·15).

**Figure 2:**
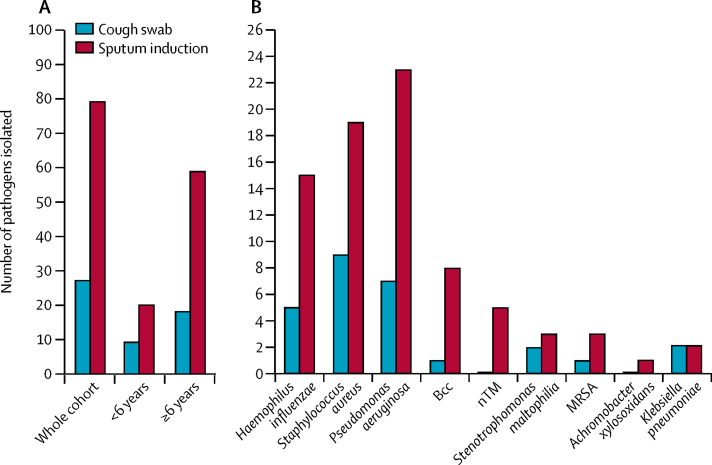
Pathogen yields from concurrent cough swab and sputum induction in 167 paired samples (A) Total pathogen yield in the whole cohort (n=167) and in subgroups of children younger than 6 years (n=62) and those aged 6 years or older (n=105). (B) Specific pathogen yields in the whole cohort (n=167). Bcc=*Burkholderia cepacia* complex. MRSA=meticillin-resistant *Staphylococcus aureus*. nTM=non-tuberculous Mycobacteria.

86 pathogens were isolated from the 167 paired cough swab and sputum-induction samples (appendix). 79 (92%) were isolated on sputum-induction samples and 27 (31%) were isolated on cough swabs (p<0·0001; figure 2A). Of the 86 pathogens isolated, 59 (69%) were identified by sputum induction only. More than one pathogen was identified on 13 (21%) of 63 positive sputum-induction samples. When analysed by age group, in children younger than 6 years, 20 (83%) of 24 pathogens were isolated on sputum induction versus nine (38%) of 24 pathogens were isolated on cough swabs (p=0·019). In children aged 6 years or older, 59 (95%) of 62 pathogens were isolated on sputum induction whereas 18 (29%) of 62 on cough swab (p<0·0001). Sputum induction identified more of almost all the specific pathogens than cough swab (figure 2B).

The additional pathogen yield (any pathogen, previously detected or not) from sputum induction compared with paired cough swab resulted in a new treatment in 52 (31%) of 167 cases. When compared with all pathogens isolated on repeated cough swabs from the preceding 12 months (median number of cough swabs six; IQR 5–7), a previously undetected pathogen was isolated in 40 (24%) of 167 sputum samples versus 15 (9%) of 167 cough swab samples (χ^2^ p=0·00039). Treatment for a previously undetected pathogen isolated exclusively on sputum induction was commenced in 32 (19%) of 167 cases.

41 of the 167 successful sputum induction and cough swab pairs from stage 1 were coupled with bronchoscopy and bronchoalveolar lavage for stage 2 (table 1). 35 patients contributed, of whom six individuals contributed twice (on two separate occasions). Median time between repeat procedures was 38 months (IQR 35–46). 32 (82%) of 41 procedures were done in symptomatic patients. Additionally, eight bronchoscopies were done on asymptomatic patients with unexplained poor spirometry tests and one bronchoscopy was done in an asymptomatic patient at the end of treatment for non-tuberculous Mycobacteria infection. Median age was 8·5 years (IQR 6·5–12·6). Six-lobe bronchoscopy was tolerated well in all patients.

At least one pathogen was isolated from at least one of the concurrent samples in 28 (68%) of the 41 pairs. 39 different pathogens were isolated (table 2). Repeated measures in individuals recruited more than once did not affect pathogen positivity in either sputum-induction samples (p=0·94) or bronchoalveolar lavage samples (p=0·75).

**Table 2:** Pathogen isolates from the paired cough swab, sputum induction, and bronchoalveolar samples

	**Cough swab**	**Sputum induction**	**Bronchoalveolar lavage sample 1 (RML)**	**Bronchoalveolar lavage sample 2 (LLi)**	**Bronchoalveolar lavage sample 3 (RLL, RUL, LLL, LUL)**
5	..	*H influenzae*	*H influenzae*	*H influenzae*	..
22*	*S aureus*	*H influenzae; S aureus; P aeruginosa*	*H influenzae; S aureus*	*H influenzae; S aureus*	*H influenzae; S aureus*
45*	..	*B cenocepacia*	..	..	..
57	..	..	*H influenzae*	*H influenzae*	*H influenzae*
60	..	*P aeruginosa*	..	..	..
70†	..	*A xylosoxidans*	..	*H influenzae*	*H influenzae; A xylosoxidans*
73†	..	*S aureus*	*S aureus*	*S aureus*	*S aureus; B multivorans*
78	..	*P aeruginosa; B multivorans*	*P aeruginosa*	*P aeruginosa; B multivorans*	*P aeruginosa; B multivorans*
79	*P aeruginosa*	..	..	..	..
80†	..	*S aureus*	*S aureus*	*S aureus*	*S aureus*
86‡	..	..	..	*M abscessus*	..
91	*S aureus; S maltophilia*	*S aureus; S maltophilia*	*S aureus*	*S aureus*	*S aureus; S maltophilia*
101†	..	..	*H influenzae; S aureus*	*H influenzae; S aureus*	*H influenzae; S aureus*
104†	*H influenzae; S aureus*	*H influenzae; S aureus; B multivorans*	*H influenzae; S aureus; B multivorans*	*H influenzae; S aureus; B multivorans*	*H influenzae; S aureus; B multivorans*
107	..	*B cepacia*	..	..	..
108*	..	*S aureus; B multivorans*	*S aureus*	*B multivorans*	*S aureus; B multivorans*
115	..	..	MRSA	MRSA	MRSA
121‡	..	*S maltophilia*	*S maltophilia*	*S maltophilia*	*S aureus; S maltophilia*
127†	..	..	..	*S maltophilia*	*S maltophilia*
134	..	MRSA	..	..	..
174*	*P aeruginosa*	*P aeruginosa*	*P aeruginosa*	*P aeruginosa*	*P aeruginosa*
178	..	..	..	..	*P aeruginosa*
179	*S aureus*	*S aureus*	*S aureus*	*S aureus*	*S aureus*
184	..	*H influenzae*	..	*H influenzae*	*H influenzae*
196	..	..	*S aureus*	*S aureus*	*S aureus*
208†	..	*M abscessus*	*M abscessus*	..	..
209	..	*P aeruginosa*	*P aeruginosa*	*P aeruginosa*	*P aeruginosa*
212	..	*H influenzae*	*H influenzae*	*H influenzae*	*H influenzae*

Of the 13 contributions that were negative with all sampling techniques (not shown), two were from patients who were asymptomatic. Of the six patients who contributed twice, one had no pathogens detected in either contributions. RML=right middle lobe. LLi=left lingular. RLL=right lower lobe. RUL=right upper lobe. LLL=left lower lobe. LUL=left upper lobe. MRSA=meticillin-resistant *Staphylococcus aureus*.

* Patients who contributed twice; other contribution was negative.

† Patients who were asymptomatic.

‡ One patient contributed twice and had different pathogens detected on the repeat procedure.

**Figure 3:**
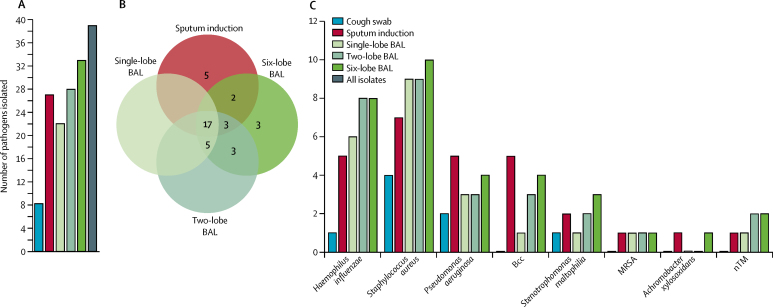
Pathogen yield for concurrent cough swab, sputum induction, and single-lobe, two-lobe, and six-lobe BAL in 41 matched samples (A) Total pathogen yield from each technique. (B) Numbers of unique and overlapping pathogen isolates for the different techniques. (C) Specific pathogen yield. BAL=bronchoalveolar lavage. Bcc=*Burkholderia cepacia* complex. MRSA=meticillin-resistant *Staphylococcus aureus*. nTM=non-tuberculous Mycobacteria.

Regarding pathogen yield from the different sampling techniques, sputum induction isolated 27 (69%) of the 39 pathogens compared with 22 (56%; p=0·092; OR 3·3, 95% CI 0·91–12·11) on single-lobe bronchoalveolar lavage, 28 (72%; p=1·0; OR 1·1, 95% CI 0·41–3·15) on two-lobe bronchoalveolar lavage, and 33 (85%; p=0·21; OR 2·2, 95% CI 0·76–6·33) on six-lobe bronchoalveolar lavage (figure 3A). Increasing numbers of pathogens were isolated on sequentially wider bronchoalveolar lavage sampling (χ^2^ p=0·023).

The sputum-induction procedure identified 17 (77%) of 22 of the pathogens present on single-lobe bronchoalveolar lavage, 20 (71%) of 28 on two-lobe bronchoalveolar lavage, and 22 (66%) of 33 on six-lobe bronchoalveolar lavage (figure 3B). Conversely, of the 27 pathogens present on sputum induction samples, single-lobe bronchoalveolar lavage isolated 17 (38%), two-lobe bronchoalveolar lavage isolated 20 (70%), and six-lobe bronchoalveolar lavage isolated 22 (80%).

For some specific pathogens, sputum induction outperformed six-lobe bronchoalveolar lavage (figure 3C). Five important pathogens (13% of total) were identified on sputum induction but not on six-lobe bronchoalveolar lavage (two *P aeruginosa* and one each of *Burkholderia cepacia, Burkholderia cenocepacia*, and MRSA). Because these pathogens were not isolated from paired cough swabs either, they are likely to be from the lower airway compartment.

Test-specific detection rates were 69% for sputum induction and 72% for two-lobe bronchoalveolar lavage, and for the combination of sputum induction with two-lobe bronchoalveolar lavage it was 90%. The test-specific detection rate for six-lobe bronchoalveolar lavage was 84%, and for the combination of sputum induction with six-lobe bronchoalveolar lavage it was 98%. These data suggest that sputum induction and bronchoalveolar lavage sample closely related, but non-identical, lower airway compartments, and that each therefore has an independent contribution in the identification of lower airway pathogens.

**Figure 4:**
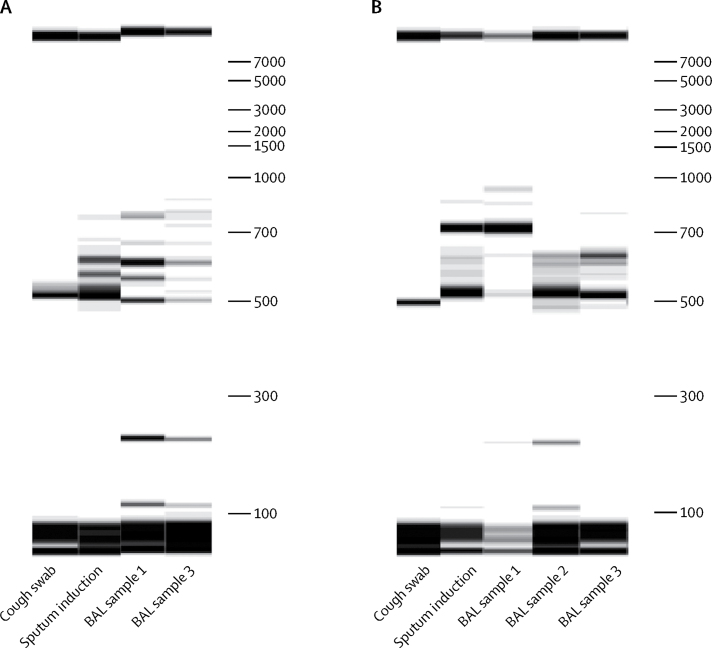
Two illustrative examples from two individuals of polymicrobial DNA signatures or RISA profiles from concurrent cough swab, sputum induction, and BAL samples (A) Example 1. (B) Example 2. BAL=bronchoalveolar lavage.

To further investigate the sampling ability of sputum induction, we extracted total DNA from cough swab, sputum induction, and bronchoalveolar lavage samples and used RISA profiling to assess the polymicrobial signatures from bacterial DNA present in those samples. In one illustrative example of RISA profiles of concurrent samples by different techniques, the sputum-induction polymicrobial signature is directly related to that of bronchoalveolar lavage, and discrete from cough swab (figure 4A). In another illustrative example, the sputum-induction polymicrobial signature is a combination of contributions from multiple bronchoalveolar lavage sample sites, indicating that sputum induction can be effective in sampling from a very wide lower airway compartment (figure 4B).

We did not want to discount those pathogens isolated by sputum induction alone as false positives, but rather, to classify them as additional lower airway pathogens. We therefore generated a sensitivity analysis against a combined gold standard consisting of all pathogens identified by sputum induction and six-lobe bronchoalveolar lavage. We defined a positive outcome as the ability to identify all pathogens in the combined gold standard, so the outcome would enable us to quantify the ability of any single approach to correctly detect all lower airway pathogens in a patient. Sensitivity of sputum induction was 0·63 (95% CI 0·42–0·79), sensitivity of two-lobe bronchoalveolar lavage was 0·59 (0·39–0·77), and sensitivity of six-lobe bronchoalveolar lavage was 0·81 (0·61–0·93). Sensitivity of combined sputum induction and two-lobe bronchoalveolar lavage was 0·93 (0·74–0·99). The ability of the sputum-induction procedure to correctly identify all lower airway pathogens in a given patient was not significantly influenced by age as a continuous variable (p=0·95) or by whether the patient was asymptomatic or symptomatic (p=0·27).

**Figure 5:**
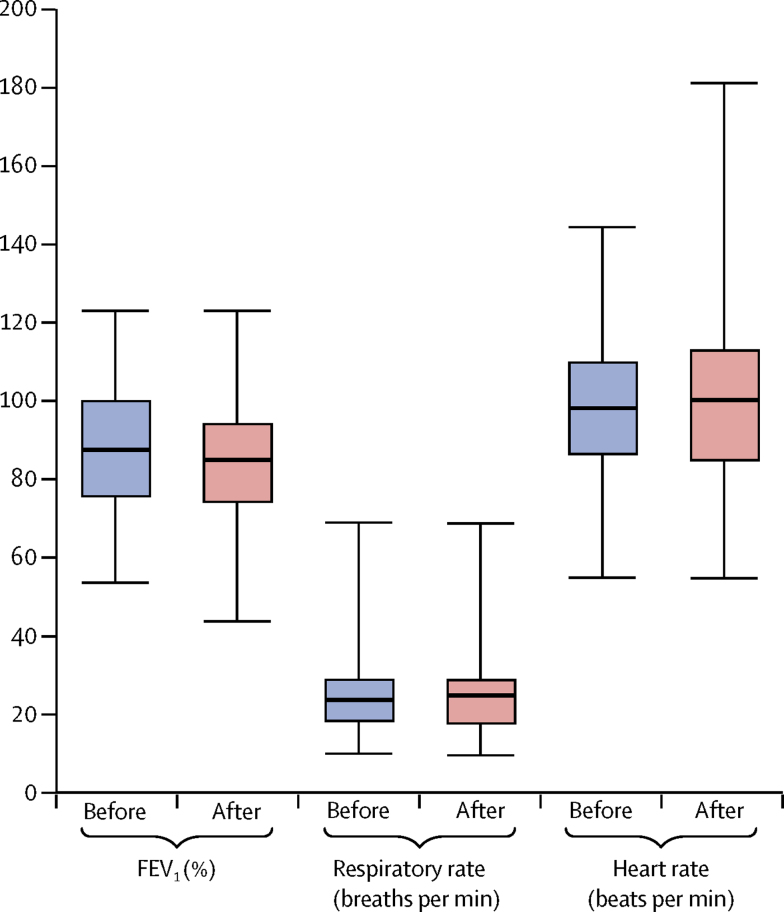
Objective assessment of tolerance to the sputum-induction procedure in 200 attempted procedures Before and after procedure measurements of FEV_1_ (where applicable), respiratory rate, and heart rate.

Objective tolerance of the sputum induction procedure was good with no significant effects on respiratory rate, heart rate, or FEV_1_% (figure 5). FEV_1_ increased by more than 5% in 21 (23%) of 90 people in whom spirometry was done. Subjective tolerance was good. Likert scales rated tolerance high, with mean parent or patient scores of 8·55 (SD 1·65) and physiotherapist scores of 9·09 (1·76).

Children in 27 (14%) of 200 sputum-induction procedures had mild side-effects: 17 (9%) became upset, of which four (2%) could not complete the procedure; six (3%) had mild wheeze, of which two (1%) could not complete the procedure; three (2%) patients vomited during oropharyngeal suction; one (<1%) became transiently dizzy. 108 (96%) of 112 (12 gave no comment) patients or parents were willing to have regular annual sputum-induction procedures.

## Discussion

In this interventional trial, we took a systematic approach to comprehensively investigate and compare bacterial sampling techniques in young children with cystic fibrosis. In particular, we tested sputum induction as an infection diagnostic technique. We compared pathogen yield from sputum induction with concurrent cough swab, single-lobe bronchoalveolar lavage, two-lobe bronchoalveolar lavage, and comprehensive six-lobe bronchoalveolar lavage to identify the relative contribution of each approach.

Most patients recruited to both stages of the study were unable to spontaneously expectorate before the sputum-induction procedure irrespective of whether they had a wet cough or not. We found the sputum-induction procedure to be well tolerated and equally successful in all age groups, in the inpatient or outpatient setting, in those who were asymptomatic or symptomatic, and in children with or without a wet cough.

In stage 1 of this study, sputum induction was compared with paired cough swab. Cough swab pathogen positivity in this study was equivalent to that reported in similar populations in other studies.^22^ Almost three times as many pathogens were identified on sputum induction compared with cough swab, and this benefit was reflected to a broadly similar degree in all age groups. More pathogens were identified on both sputum induction and cough swab in children aged 6 years or older, reflecting the greater pathogen prevalence in the older age group. The benefits of sputum induction over cough swab were seen in symptomatic and asymptomatic children, supporting the use of sputum induction over cough swab in all situations. Sputum induction was positive for a pathogen in 38% of paired samples, positive for a pathogen not identified on paired cough swab in 31% of cases, and positive for a new pathogen not isolated on repeated cough swabs from the preceding 12 months in 24% of cases. Sputum induction had a considerable effect on patient care, with new treatment implemented as a consequence in 31% of cases.

In stage 2 of the study, sputum induction was compared with single-lobe, two-lobe, and six-lobe bronchoalveolar lavage in a group of children who were largely symptomatic. Sequentially higher proportions of pathogens were detected by single-lobe, two-lobe, and six-lobe bronchoalveolar lavage. The proportion of pathogens isolated by sputum induction and two-lobe bronchoalveolar lavage were largely equivalent. Six-lobe bronchoalveolar lavage only identified 85% of pathogens isolated from all approaches, since some important pathogens were uniquely isolated on sputum induction. Patient age did not affect the ability for sputum induction to correctly describe all lower airway pathogens.

By using multi-approach concurrent sampling from upper and lower airways in the same patient, we estimated whether pathogens identified by sputum induction were upper or lower airway residents. A large proportion of pathogens identified by sputum induction were identified on bronchoalveolar lavage, confirming that sputum induction does effectively sample the lower airway. We found that with sequentially wider bronchoalveolar lavage sampling, more pathogens that were isolated on sputum induction were also identified on bronchoalveolar lavage, indicating that sputum induction is capable of sampling very widely from the lower airway. Using DNA RISA profiling we showed that sputum induction can capture the diversity of bacteria associated with multiple bronchoalveolar compartments.

A proportion of pathogens (13%) were identified on sputum induction but not on six-lobe bronchoalveolar lavage or cough swabs. With the assumption that concurrent cough swab and bronchoalveolar lavage samples were true negatives,^23^ this finding suggests that sputum induction can identify pathogens from compartments of the respiratory tract not sampled by the other methods, and raises the question as to where these pathogens reside. Bacterial bronchitis is common in young children with cystic fibrosis who are symptomatic, suggesting the predominant focus of acute infection might often be the large intrathoracic airways rather than the alveolar bed. The large intrathoracic airways together constitute a lower-airway compartment that is most easily sampled by the sputum-induction procedure, is largely bypassed by even the most extensive approach to bronchoalveolar lavage, and is a compartment perhaps inadequately considered in the current paradigm of lower airway sampling. In this study, sputum induction was more successful at isolating the important Gram-negative pathogens, *P aeruginosa* and *B cepacia* complex species, compared with concurrent six-lobe lavage (figure 3C). This raises the possibility that for some pathogens the large intrathoracic airways might be the optimal lower airway environment for early infection.

We generated a sensitivity analysis against a combined gold standard consisting of all pathogens identified by sputum induction and six-lobe bronchoalveolar lavage. Sputum induction is shown in these data to be marginally more sensitive than the current gold-standard two-lobe bronchoalveolar lavage (0·63 *vs* 0·59). Six-lobe bronchoalveolar lavage has a higher sensitivity at 0·81. However, sputum induction and two-lobe bronchoalveolar lavage combined have a sensitivity of 0·93. These data highlight independent, sizeable gains in pathogen detection from both sputum induction and extended six-lobe bronchoalveolar lavage, over and above the current gold-standard two-lobe bronchoalveolar lavage. This in turn questions whether two-lobe bronchoalveolar lavage can be considered adequate as a standalone approach to understanding lower airway microbiology. We advocate from the present data that sputum induction and six-lobe lavage should be done together as a new standard of care for comprehensive assessment of the lower airway pathogen environment in children with cystic fibrosis.

The present data also support sputum induction as a non-invasive surrogate for bronchoalveolar lavage. We propose that in symptomatic patients, bronchoscopy and bronchoalveolar lavage should be reserved for those who have not responded to appropriate or empirical antibiotic treatment and whose sputum-induction cultures do not explain the persistence of symptoms. From our data, a successful sputum-induction sample taken before bronchoscopy and six-lobe bronchoalveolar lavage correctly identified all lower airway pathogens in 63% of patients who had one or more pathogen present. The routine use of sputum induction followed by appropriate treatment for those pathogens isolated could therefore substantially reduce the need for bronchoscopy in symptomatic patients with cystic fibrosis. This has notable implications both for quality of care and cost.

Limitations of the study include the fact that study investigators were not blinded to outcome, recruitment was not randomised, and some patients contributed on more than one occasion. However, the study is likely to be representative because 70% of the patients who attend the South, West, and Mid-Wales Children's Cystic Fibrosis Service were recruited into this study. We adjusted statistical outcomes for repeated measures in those patients that were recruited on more than one occasion. The bronchoalveolar lavage data apply to a cohort of patients who were largely symptomatic, because patients were recruited when bronchoscopy was clinically indicated. The outcomes of stage 2 of the study therefore relate to symptomatic patients and might not be directly applicable to surveillance programmes in asymptomatic patients with cystic fibrosis.

In conclusion, we have established sputum induction as superior to cough swab, and as a credible approach to sampling the lower airway in symptomatic children with cystic fibrosis. We showed benefit in patients of all ages, and in those who are unable to spontaneously expectorate. We suggest that the large intrathoracic airways constitute an important lower airway compartment that is inadequately sampled by standard approaches to pathogen surveillance in children with cystic fibrosis. We have shown that both sputum induction and six-lobe bronchoalveolar lavage contribute important independent gains in pathogen detection compared with the current gold-standard two-lobe bronchoalveolar lavage, and advocate sputum induction and six-lobe bronchoalveolar lavage combined as a new standard of care in the assessment of the lower airway pathogen environment in children with cystic fibrosis. In symptomatic patients, doing sputum induction before bronchoalveolar lavage will correctly describe the lower airway pathogen environment in almost two-thirds of patients, and if used routinely, could substantially reduce the number of bronchoscopy procedures required. We recommend implementation of sputum induction as a regular contribution to cystic fibrosis care in children. This recommendation is supported by the tolerability of the procedure in all age groups, the ease of repeatability, the acceptability to parents and patients, the high success of obtaining samples, the high proportion of pathogens identified, the applicability to both the inpatient and outpatient setting, and the clear economic savings compared with bronchoscopy.
