# Supplementary material for: The CF-Sputum Induction Trial (CF-SpIT) to assess lower airway bacterial sampling in young children with cystic fibrosis: a prospective internally controlled interventional trial
Source: Lancet Respir Med. 2018 Jun;6(6):461–71. doi: 10.1016/S2213-2600(18)30171-1 (PMC5971213; doi:10.1016/S2213-2600(18)30171-1)
Supplement: Supplementary appendix [file mmc1.pdf]

THE LANCET
Respiratory Medicine

Supplementary appendix

This appendix formed part of the original submission and has been peer reviewed. We post it as supplied by the authors.

Supplement to: Ronchetti K, Tame J-D, Paisey C, et al. The CF-Sputum Induction Trial (CF-SpIT) to assess lower airway bacterial sampling in young children with cystic fibrosis: a prospective internally controlled interventional trial. *Lancet Respir Med* 2018; published online May 16. http://dx.doi.org/10.1016/S2213-2600(18)30171-1.

On-line appendix

CF-SpIT Research Protocol for sputum-induction sampling

- Sputum induction should be performed after a 2 hour fast.
- Make an initial assessment of the chest. Attach an oxygen saturation monitor and document oxygen saturations, heart rate, respiratory rate. Perform lung function in children older than 7 years old.
- Before the procedure, obtain a cough swab.
- Administer 200mcg salbutamol via metered dose inhaler and spacer to prevent bronchospasm.
- Use a jet nebuliser attached to wall oxygen at a flow rate of 5 l/min to deliver 8 ml of 7% sterile hypertonic saline for 15 minutes.
- Make an assessment of the chest every 5 minutes.
- Apply physiotherapy techniques during and after procedure, including chest percussion, vibration, active cycle of breathing techniques, PEP and assisted autogenic drainage.
- Obtain sputum either by expectoration (in children able to cooperate) or by suctioning through the nasopharynx or oropharynx using a sterile, mucus extractor or suction catheter size 6.
- Make a final assessment and document observations, oxygen saturations, and lung function in children over 7 years old.

DNA extraction

All samples were centrifuged at 14 800 rpm in a microcentrifuge for 5 min and reduced to a volume of 400 µL by pipetting off the supernatant. Highly viscous sputum was pretreated with 1:1 volume ratio of 4M guanidium thiocyanate to enable pipetting and enhance extraction. Total nucleic acids were extracted from all reduced samples using the automated Maxwell 16 DNA purification system and Tissue DNA kit (Promega) in accordance with the manufacturer's instructions. Extracted DNA samples were stored at –80°C until further analysis.

Ribosomal Intergenic Spacer Analysis (RISA)

Briefly, PCRs were set up with 5 µL of extracted sputum DNA and 10 pmol of each RISA PCR primer (1406F, TGYACACACCGCCCGT, and 23SR, GGGTTBCCCCATTCRG). RISA amplicons (2 µL of amplified DNA) were separated by microfluidics (Agilent 2100 Bioanalyzer; Agilent Technologies, Santa Clara, CA, USA), and their profiles were analysed using Gelcompar II (Applied Maths, SintMartens Latem, Belgium). DNA from pure bacterial cultures of reference cystic fibrosis pathogen species was used to generate control ITS amplicons for putative pathogen identification based on size correlation. Negative controls containing water were included with every batch of sputum DNA.

Table : Bacterial isolates from 167 paired cough swab and sputum-induction samples

EVENT NUMBER CF-SpIT	DATE	COUGH SWAB	SPUTUM INDUCTION
1	23/01/2012		failed
2	26/01/2012	P.aeruginosa	P.aeruginosa
3	02/02/2012		
5	22/02/2012		H.influenzae
6	28/02/2012		failed
7	27/03/2012		E.coli
8	30/03/2012		M.abscessus
9	03/04/2012		Phyllobacterium species
11	17/04/2012		
12	23/04/2012	P.aeruginosa	P.aeruginosa
13	24/04/2012		
14	22/05/2012		
15	22/05/2012		B.multivorans
16	11/06/2012	E.coli	failed
17	14/06/2012		P.aeruginosa
18	18/06/2012		
19	22/06/2012		S.aureus
20	28/06/2012		failed
21	10/07/2012		P.aeruginosa, S. marcescens, MAI
22	17/07/2012	S.aureus	S.aureus, H.influenzae, P.aeruginosa
23	26/07/2012		
24	06/08/2012		failed
25	14/08/2012		
26	28/08/2012		failed
27	18/09/2012		
28	25/09/2012		failed
30	09/10/2012		P.aeruginosa
31	23/10/2012	S.aureus	
32	25/10/2012		
33	29/10/2012		S.aureus
34	30/10/2012		
35	30/10/2012		
36	01/11/2012		
37	07/11/2012	S.aureus	S.aureus, S.maltophilia
38	27/11/2012		
39	11/12/2012		
40	07/01/2013		
41	08/01/2013		
42	04/02/2013		failed
43	05/02/2013		

EVENT NUMBER CF-SpIT	DATE	COUGH SWAB	SPUTUM INDUCTION
44	11/02/2013	B.multivorans	B. multivorans
45	28/02/2013		B.cenocepacia
46	27/03/2013		failed
47	27/03/2013	H.influenzae	H.influenzae
48	10/04/2013		
49	10/04/2013		
50	17/04/2013		
51	30/04/2013		
52	07/05/2013		failed
53	09/05/2013		
54	15/05/2013		
55	20/05/2013		failed
56	21/05/2013		failed
57	06/06/2013		E. Cloacae
58	24/06/2013		P.aeruginosa
59	25/06/2013		failed
60	01/07/2013		P.aeruginosa
61	20/08/2013		failed
63	12/09/2013	S.aureus, K.pneumoniae, E. asburiae	S.aureus, K.pneumoniae
64	16/09/2013		
65	25/09/2013		P.aeruginosa
66	27/09/2013		S. marcescens
67	27/09/2013		
68	30/09/2013		
69	08/10/2013		
70	09/10/2013		A. xylosoxidans, E.coli
71	18/10/2013		P.aeruginosa
72	23/10/2013		K.pneumoniae
73	25/10/2013		S.aureus
75	25/11/2013		M.abscessus
78	06/01/2014		P.aeruginosa, B.multivorans, E.Asburiae
79	16/01/2014	P.aeruginosa	
80	22/01/2014		S.aureus
83	18/03/2014		
84	18/03/2014		
85	19/03/2014		
86	25/03/2014		
87	07/04/2014		
89	30/04/2014		
90	18/07/2014		
91	21/07/2014	S.aureus, S.maltophilia	S.aureus, S.maltophilia
92	13/08/2014		S.aureus
93	20/08/2014	P.aeruginosa	P.aeruginosa

EVENT NUMBER CF-SpIT	DATE	COUGH SWAB	SPUTUM INDUCTION
94	27/08/2014		
95	16/09/2014		S. marcescens
96	16/09/2014		S. marcescens
97	25/11/2014		
98	12/01/2015		
99	06/02/2015		
101	03/03/2015		
103	10/04/2015		
104	16/04/2015	H.influenzaeS.aureus	H.influenzae, S.aureus, B.multivorans
105	28/05/2015		
106	02/06/2015		
107	06/02/2015		B.cepacia
108	25/06/2015		S.aureus, B.vietnamenisis
109	30/06/2015		
110	30/06/2015	serratia	S.marcescens
111	30/06/2015		failed
112	07/07/2015	H.influenzae	H.influenzae
113	07/07/2015		failed
114	14/07/2015		
115	16/07/2015		
116	21/07/2015	PSS	
117	21/07/2015	MRSA	MRSA, H.influenzae
118	28/07/2015		H.influenzae
119	04/08/2015		
120	11/08/2015		failed
121	13/08/2015		S.maltophilia
122	18/08/2015		
123	20/08/2015		
124	25/08/2015		
125	27/08/2015		
126	01/09/2015	S.aureus	S.aureus, E.coli, Exophalia
127	17/09/2015		
128	22/09/2015		
129	22/09/2015		H.influenzae, S.aureus, Pseudomonas sp
130	06/10/2015		failed
131	06/10/2015		P.aeruginosa, B.multivorans
132	13/10/2015		
133	13/10/2015		
134	14/10/2015		MRSA
135	20/10/2015		
136	20/10/2015		
137	27/10/2015		P.aeruginosa
138	02/10/2015		P.aeruginosa

EVENT NUMBER CF-SpIT	DATE	COUGH SWAB	SPUTUM INDUCTION
139	10/11/2015		S.aureus, P.aeruginosa, A. Fumigatus, Exophiala
140	17/11/2015	klebsiella	H.influenzae
141	24/11/2015		failed
142	01/12/2015		failed
143	15/12/2015		failed
144	12/01/2016		H.influenzae
145	12/01/2016		
146	19/01/2016	E.coli	E.coli, H.influenzae, S.aureus
147	21/01/2016	S.aureus	S.aureus
149	28/01/2016		failed
150	02/02/2016		H.influenzae
151	02/02/2016		
152	09/02/2016	P.aeruginosa	
153	09/02/2016	S. marcescens	S. Marcescens
154	16/02/2016		
155	16/02/2016		
156	23/02/2016		S.aureus
157	19/02/2016		
158	10/03/2016		
159	15/03/2016	H.influenzae	
160	29/03/2016		
161	05/04/2016	E.coli	E.coli
162	05/04/2016		failed
163	05/04/2016		
165	19/04/2016		P.aeruginosa
166	26/04/2016		
167	03/05/2016		
168	10/05/2016	A. Fumigatus	
169	18/05/2016		failed
170	17/05/2016		failed
171	23/05/2016		
172	24/05/2016	H.influenzae	H.influenzae
173	24/05/2016		
174	26/05/2016	P.aeruginosa	P.aeruginosa
175	14/07/2016		failed
176	19/07/2016		P.aeruginosa
177	19/07/2016		P.aeruginosa
178	21/07/2016		
179	25/07/2016	S.aureus	S.aureus
180	26/07/2016		failed
182	02/08/2016		failed
183	02/08/2016		failed
184	11/08/2016		H.influenzae

EVENT NUMBER CF-SpIT	DATE	COUGH SWAB	SPUTUM INDUCTION
186	30/08/2016		P.aeruginosa
188	27/09/2016		
189	12/10/2016		
190	19/10/2016		S.aureus
191	21/10/2016		P.aeruginosa,
192	04/10/2016		
193	13/10/2016		failed
194	27/10/2016		
195	01/11/2016		
196	02/11/2016		lightgryeast
197	15/11/2016		A.fumigatus
198	06/12/2016		
199	08/12/2016	P.aeruginosa	E.cloacae
200	13/12/2016		
201	20/12/2016		
202	31/01/2017		failed
203	31/01/2017		M. abscessus
204	07/02/2017	S.maltophilia	failed
205	07/02/2017		
206	09/02/2017		
207	14/02/2017	S.maltophilia	
208	14/02/2017		M. abscessus
209	23/02/2017		
211	07/03/2017		failed
212	08/03/2017		E.coli, H.influenzae
213	21/03/2017		
214	23/03/2017		
216	04/07/2017		
217	05/07/2017		P.aeruginosa
218	04/07/2017		MRSA
219	04/07/2017		
